# Association between physical activity and cancer risk among Chinese adults: a 10-year prospective study

**DOI:** 10.1186/s12966-022-01390-1

**Published:** 2022-12-12

**Authors:** Jian Su, Yuchen Jiang, Xikang Fan, Ran Tao, Ming Wu, Yan Lu, Yujie Hua, Jianrong Jin, Yu Guo, Jun Lv, Pei Pei, Zhengming Chen, Liming Li, Jinyi Zhou

**Affiliations:** 1 Department of Noncommunicable Chronic Disease and Prevention, Jiangsu Provincial Center for Disease Control and Prevention, Nanjing, 210009 China; 2 Department of Epidemiology, School of Public Health, Nanjing Medical University, Nanjing, 211166 China; 3 Department of Noncommunicable Chronic Disease Control and Prevention, Suzhou City Center for Disease Control and Prevention, Suzhou, 215004 China; 4 Wuzhong District Center for Disease Control and Prevention, Suzhou, 215100 China; 5 Fuwai Hospital Chinese Academy of Medical Sciences, Beijing, 102308 China; 6 Department of Epidemiology and Biostatistics, School of Public Health, Peking University, Beijing, 100191 China; 7 Center for Public Health and Epidemic Preparedness and Response, Peking University, Beijing, 100191 China; 8 Chinese Academy of Medical Sciences, Beijing, 102308 China; 9 Clinical Trial Service Unit and Epidemiological Studies Unit, Nuffield Department of Population Health, University of Oxford, Oxford, OX3 7LF UK

**Keywords:** Physical activity, Cancer, Prospective cohort study

## Abstract

**Background:**

In China, the quantity of physical activity differs from that in Western countries. Substantial uncertainty remains about the relevance of physical activity for cancer subtypes among Chinese adults.

**Objective:**

This study aimed to investigate the association between total daily physical activity and the incidence of common types of cancer.

**Methods:**

A total of 53,269 participants aged 30–79 years were derived from the Wuzhong subcohort of the China Kadoorie Biobank study during 2004–2008. We included 52,938 cancer-free participants in the final analysis. Incident cancers were identified through linkage with the health insurance system and death registries. Cox proportional hazard models were introduced to assess the associations of total daily physical activity with the incidence of 6 common types of cancer.

**Results:**

During a follow-up of 10.1 years, 3,674 cases of cancer were identified, including 794 (21.6%) from stomach cancer, 722 (19.7%) from lung cancer, 458 (12.5%) from colorectal cancer, 338 (9.2%) from liver cancer, 250 (6.8%) from breast cancer, and 231 (6.3%) from oesophageal cancer. Compared to the participants in the lowest quartile of physical activity levels, those in the highest quartile had an 11% lower risk for total cancer incidence (hazard ratio [HR]: 0.89, 95% confidence interval [CI]: 0.81–0.99), 25% lower risk for lung cancer incidence (HR: 0.75, 95% CI: 0.60–0.94), and 26% lower risk for colorectal cancer incidence (HR: 0.74, 95% CI: 0.55–1.00). There were significant interactions of physical activity with sex and smoking on total cancer (both *P* for interaction < 0.005), showing a lower risk for females and never smokers (HR: 0.92, 95% CI: 0.87–0.98 and HR: 0.93, 95% CI: 0.87–0.98, respectively).

**Conclusions:**

Higher physical activity levels are associated with a reduced risk of total, lung, and colorectal cancer.

**Supplementary Information:**

The online version contains supplementary material available at 10.1186/s12966-022-01390-1.

## Introduction

Cancer is one of the leading causes of morbidity and mortality worldwide. According to GLOBOCAN estimates, there were an anticipated 19.3 million new cancer cases and nearly 10.0 million cancer deaths worldwide in 2020, of which China accounted for 23.7% of the total cancer cases and 30.2% of the cancer deaths [1]. Physical activity is known to reduce the risk of cancer in Western countries [2, 3], as well as the risk of heart disease and all-cause mortality [4–6]. Less is known, however, about whether physical activity reduces the risk of cancer in China. Physical inactivity is highly prevalent, with an estimated 31.0% of people in China and worldwide not attaining the recommended physical activity levels [7]. Thus, it is of major public health importance to establish an evidence base of physical activity associated with cancer risk to inform cancer prevention policies.

Total physical activity is composed of occupational, commuting, household, and leisure-time physical activity. In China, there has been a substantial shift from labour-intensive lifestyles to more sedentary lifestyles in recent decades [8]. Several previous studies in China have examined the associations of physical activity with cancer, but they were constrained by analyses restricted to a single sex [9] or specific domains of physical activity [10, 11]. Several meta-analyses reduced these limitations by pooling published research [12, 13]. Pooled studies, on the other hand, have often been heterogeneous in terms of research design (e.g., case–control vs. prospective cohort), physical activity types evaluated (e.g., leisure-time vs. occupational activity), and contrasts examined (e.g., tertiles vs. quintiles) [13–15]. Risk estimates can be weakened by such heterogeneity and hide genuine underlying connections.

The aims of the present study were (1) to quantify the associations of total physical activity with the risk of common types of cancer and (2) to examine whether these associations differed by sex, age, smoking status and body mass index (BMI).

## Methods

### Study population

Detailed information about the China Kadoorie Biobank (CKB) study design, survey methods and participants’ characteristics have been described elsewhere [16–18]. The data utilized in the current study were obtained from the Wuzhong District of Suzhou city, one of the 10 regions included in the CKB study. In brief, 53,269 participants aged 30–79 years were recruited for the baseline survey between June 2004 and July 2008.

In this study, we excluded participants who had been diagnosed with malignant cancer (excluding nonmelanoma skin cancer) before baseline (*n* = 331). After this exclusion, a total of 52,938 (22,234 men, 30,704 women) participants remained for inclusion in the final analyses.

### Assessment of physical activity

Details of the methods used to assess physical activity have been previously reported [18, 19]. At the baseline survey, participants were asked about the intensity, frequency and duration of physical activities (including occupation, commuting, housework and leisure-time activity) during the past 12 months. Metabolic equivalents of tasks (METs) of different types of activities were adopted from the 2011 Compendium Of Physical Activities [20]. The MET of each activity was multiplied by the frequency and duration of physical activity to calculate physical activity in MET-hours per day (MET-h/day) from each activity. Occupational physical activity included all physical activity performed during paid employment, and nonoccupational physical activity included all physical activity performed during travel to and from work, household activity and leisure-time exercise. Total physical activity was the summation of occupational and nonoccupational physical activity.

### Assessment of covariates

Covariate information was collected in the baseline questionnaire, including sociodemographic characteristics (age, sex, level of education, and marital status), lifestyle behaviours (alcohol consumption, smoking status, consumption of fresh fruit and red meat). For alcohol consumption, we asked about drinking frequency (‘Never regular drinker’, ‘Ex regular drinker’, ‘Occasional or seasonal drinker’, ‘Monthly drinker’, ‘Reduced intake drinker’, ‘Weekly drinker’). For smoking status, we asked about smoking status (‘Never smoker’, ‘Occasional smoker’, ‘Ex regular smoker’, ‘Smoker’). For consumption of fresh fruit and red meat, we asked about consumption of fresh fruit and red meat (‘Daily’, ‘4–6 days per week’, ‘1–3 days per week’, ‘Monthly’, ‘Never/rarely’). Baseline measurements of body weight and height were measured by trained staff using well-calibrated instruments. Body mass index was calculated as weight in kilograms divided by height in metres squared.

### Ascertainment of outcomes

Incident outcome cases since the participants’ enrolment into the cohort were identified utilizing linkage with local disease and death registries, the national health insurance system, and by active follow-up [21]. Approximately 98% of participants were covered by the health insurance system, which recorded details of all episodes of hospitalization and coded examination and treatment procedures. The 10^th^ revision of the International Classification of Diseases (ICD-10) was used to code the incident events by trained staff “blinded” to baseline information. In this study, we selected cancers with an incidence of 200 cases or more, including total cancer cases coded as C00-C99, oesophageal cancer [C15], stomach cancer [C16], colorectal cancer [C18-C20], liver cancer [C22], lung cancer [C33-C34] and breast cancer [C50].

### Statistical analysis

Total daily physical activity was categorized into four groups based on quartiles among 52,938 participants. Mean values and prevalence of baseline characteristics were calculated for categories of total physical activity at baseline. Continuous variables were described as means (standard deviations, SDs), and categorical variables were described as proportions (%).

Hazard ratios and 95% confidence intervals (CIs) for the incidence of common types of cancer associated with total physical activity levels were estimated using Cox proportional hazard regression models. Tests for trend were assessed by including physical activity as a continuous variable. Physical activity was also modelled as a continuous variable to estimate the risk associated with a standard deviation (SD) higher level of physical activity. The Cox regression analyses were stratified by age in 5-year intervals to fit proportional hazard assumption, and adjusted for sex (female, male), level of education (no formal schooling, middle school and below, or high school and above), marital status (married, widowed, separated or divorced or never married), alcohol consumption (never regular drinker, former regular drinker, occasional drinker, or regular drinker), smoking status (never regular smoker, former regular smoker, occasional smoker, or regular smoker), consumption of fresh fruit (daily, 4–6 days per week, 1–3 days per week, monthly, never or rarely) and red meat (≥ 4 days per week, 1–3 days per week, monthly or less), and BMI (continuous). The linearity of physical activity and cancer associations was evaluated with restricted cubic splines.

We also examined the associations of total physical activity with the incidence of total cancer, lung cancer and colorectal cancer among prespecified baseline subgroups based on age (< 60, ≥ 60 years), sex, BMI (< 25, ≥ 25 kg/m^2^) and smoking status (never, ever). To investigate potential interaction effects, we used a likelihood ratio test comparing the models with and without a cross-product term between total physical activity levels and each of the stratification variables.

Furthermore, several sensitivity analyses were conducted to test the robustness of the results excluding cases of cancer diagnosed during the first two years of follow-up or excluding individuals with poor self-rated health at baseline. All analyses used two-sided *P* values and were conducted using R V4.1.3.

## Results

### Baseline characteristics of participants by physical activity

Among the 52,938 participants, the mean (SD) age at baseline was 52.1 (10.3) years, the mean (SD) BMI was 24.0 (3.2) kg/m^2^, 58.0% were women and 25.1% had a family history of cancer. A total of 3,674 cases of incident cancers were identified, including 794 cases of stomach cancer, 722 cases of lung cancer, 458 cases of colorectal cancer, 338 cases of liver cancer, 250 cases of breast cancer, and 231 cases of oesophageal cancer (Supplementary Table 1). Compared with individuals with lower levels of physical activity, those with higher levels of physical activity were more likely to be male and younger. Such individuals also had lower levels of BMI and higher levels of education (Table 1).

**Table 1:** Baseline characteristics of 52,938 participants by level of total daily physical activity^a^

Baseline characteristics	Overall (*N* = 52,938)	Physical activity			
		Q1	Q2	Q3	Q4
Age, year	52.1 ± 10.3	58.5 ± 10.4	52.0 ± 10.3	49.1 ± 9.0	48.6 ± 8.4
Female, %	58.0	62.4	65.0	56.2	48.4
Middle school and above, %	37.8	29.5	40.8	41.9	38.9
Married, %	92.7	87.6	92.8	94.9	95.6
Alcohol intake, %					
Never regular drinker	58.8	66.0	62.5	55.9	50.6
Former regular drinker	2.1	3.6	1.4	1.5	1.8
Occasional drinker: < once/week	21.7	17.5	21.5	23.8	23.8
Regular drinker: ≥ once/week	17.5	12.8	14.5	18.8	23.9
Smoking status, %					
Never regular smoker	60.8	65.8	67.3	58.6	51.4
Former regular smoker	5.5	7.9	4.4	4.7	5.3
Occasional smoker	4.7	3.7	4.4	5.1	5.7
Regular smoker	28.9	22.6	23.9	31.6	37.7
Fresh fruit consumption, %					
Daily	17.7	19.1	20.4	17.5	13.7
4–6 days per week	11.1	10.1	12.8	11.0	10.3
1–3 days per week	35.0	32.1	34.8	36.5	36.6
Monthly	31.5	31.7	28.4	30.7	35.1
Never/rarely	4.8	6.9	3.6	4.3	4.3
Red meat consumption, %					
4–7 days per week	44.7	33.1	44.0	49.0	52.7
1–3 days per week	47.8	55.2	48.5	45.1	42.2
Monthly/never/rarely	7.6	11.7	7.5	5.9	5.1
BMI, kg/m^2^	24.0 ± 3.2	24.1 ± 3.4	24.1 ± 3.2	24.0 ± 3.1	23.9 ± 3.0
Family history of cancer, %^b^	25.1	26.1	24.6	24.6	25.1

BMI: Body mass index

^a^Level of total physical activity was divided into four groups by quartiles, with Q1 as the lowest quartile group. Baseline characteristics were presented as the mean (SD) or percentage

^b^Based on self-reported cancer in father, mother or siblings

### Association of total physical activity with cancer risk

Total physical activity was inversely associated with the risk of total cancer, with adjusted HRs of 1.00, 0.96 (95% CI: 0.88, 1.05), 0.88 (95% CI: 0.80, 0.97) and 0.89 (95% CI: 0.81, 0.99) from the lowest to the highest group (*P*_trend_ = 0.009; Table 2). Each 1 SD (15.2 MET-h/d) higher baseline total physical activity was associated with a 5% lower risk of total cancer (HR: 0.95, 95% CI: 0.92, 0.98). We observed similar inverse associations of total physical activity with the risk of lung cancer and colorectal cancer. Compared with participants in the lowest level of physical activity group, those in the highest level of activity group showed a 25% reduction in the risk of lung cancer (HR: 0.75, 95% CI: 0.60, 0.94) and a 26% reduction in the risk of colorectal cancer (HR: 0.74, 95% CI: 0.55, 1.00). There were suggestive associations with stomach cancer (HR: 0.95, 95% CI: 0.77, 1.18), liver cancer (HR: 0.82, 95% CI: 0.58, 1.15), oesophageal cancer (HR: 0.84, 95% CI: 0.56, 1.27), and breast cancer (HR: 0.87, 95% CI: 0.56, 1.33) (Table 2). We evaluated the linearity of physical activity and total, colorectal, lung cancer associations by restricted cubic splines. Associations were predominantly linear (Supplementary Fig. 1).

**Table 2:** Associations between daily total physical activity and cancer incidence among 52,938 participants

Cause of incidence	Physical activity				*P-*trend	per 1-SD increment
	Q1	Q2	Q3	Q4		
Total Cancers						
No of events	1263	868	694	710		
HR (95% CI)	1.00	0.96 (0.88, 1.05)	0.88 (0.80, 0.97)	0.89 (0.81, 0.99)	0.009	0.95 (0.92, 0.98)
Stomach Cancer						
No of events	281	163	157	157		
HR (95% CI)	1.00	0.93 (0.77, 1.14)	1.02 (0.82, 1.25)	0.95 (0.77, 1.18)	0.957	0.99 (0.92, 1.08)
Lung Cancer						
No of events	279	165	121	135		
HR (95% CI)	1.00	0.86 (0.71, 1.06)	0.71 (0.57, 0.90)	0.75 (0.60, 0.94)	0.004	0.89 (0.82, 0.96)
Colorectal Cancer						
No of events	175	124	68	75		
HR (95% CI)	1.00	1.03 (0.81, 1.31)	0.67 (0.50, 0.90)	0.74 (0.55, 1.00)	0.007	0.86 (0.77, 0.96)
Liver Cancer						
No of events	120	76	72	60		
HR (95% CI)	1.00	1.04 (0.77, 1.40)	1.08 (0.78, 1.48)	0.82 (0.58, 1.15)	0.364	0.95 (0.84, 1.06)
Oesophagus Cancer						
No of events	86	47	39	45		
HR (95% CI)	1.00	0.92 (0.63, 1.33)	0.82 (0.55, 1.24)	0.84 (0.56, 1.27)	0.384	0.94 (0.81, 1.08)
Breast Cancer (female only)						
No of events	56	67	52	41		
HR (95% CI)	1.00	1.08 (0.75, 1.56)	0.94 (0.63, 1.41)	0.87 (0.56, 1.33)	0.265	0.91 (0.78, 1.07)

Multivariate models were stratified by age (5 years intervals) and adjusted for: sex, education, marital status, alcohol intake, smoking status, fresh fruit intake, red meat intake, and BMI

### Subgroup analyses

**Fig. 1:**
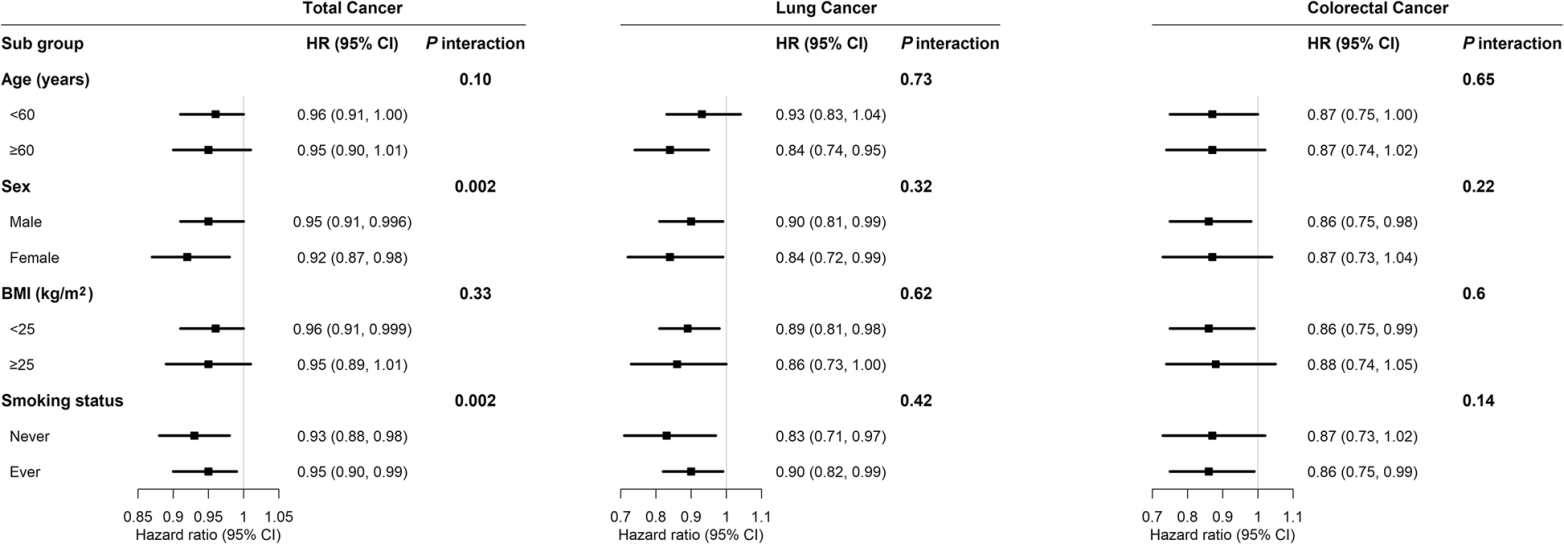
Subgroup analysis of associations between daily total physical activity and cancer incidence

The strength of the inverse association of total physical activity was generally similar across subgroups stratified according to age and BMI. Significant differences across strata were observed for sex (*P* = 0.002 for interaction) and smoking status (*P* = 0.002 for interaction) among participants diagnosed with cancer, with a stronger inverse association among female participants and participants who had never smoked (Fig. 1).

### Sensitivity analyses

The associations of total physical activity with the risk of total cancer, lung cancer and colorectal cancer were not changed substantially in sensitivity analyses, either with the exclusion of participants diagnosed during the first two years of follow-up or the exclusion of participants with poor self-rated health (Supplementary Tables 2–3).

## Discussion

In this Chinese population, a high level of total physical activity was inversely associated with several subtypes of cancer. The inverse associations of total physical activity were attenuated by 11% for total cancer risk, 25% for lung cancer risk and 26% for colorectal cancer risk. Subgroup analysis indicated that for total cancer, this inverse association was more pronounced among women and non-smokers.

Prospective cohort studies in Western populations reported an inverse association for total cancer incidence [22, 23]. A cohort study of 521,330 participants and 36,994 cancer cases in 10 European countries reported that a higher level of physical activity was associated with a 4% (95% CI: 1%, 6%) lower risk of cancer [23]. In this study, the finding for total physical activity was consistent with previous studies. For lung cancer, a meta-analysis of 20 cohort studies with 31,807 cases showed a 17% (95% CI: 10%, 23%) lower risk comparing high and low categories of physical activity with no significant differences between subgroups [24]. The findings in our study were consistent with those of prior studies. However, a new genetic analysis found little evidence that a higher level of physical activity would help prevent lung cancer [25]. Nevertheless, this study only included individuals of European ancestry; additionally, the risk factor and outcome samples came from different populations. Meanwhile, the results of previous MR studies suggested that this association is tentatively inconclusive [25–27]. Considering the low explanation of physical activity status by genes and the reduced risk of physical activity for total cancer in this study, future RCT studies focused on the association between physical activity and lung cancer may provide solid evidence on this issue. For colorectal cancer, a meta-analysis of 19 cohort studies in the USA, Europe and Japan with 53,929,648 total person-years of follow-up reported an inverse association between physical activity and colorectal cancer [28]. In this study, the finding was consistent with previous studies. In addition, previous MR studies also supported the conclusion of this study [29, 30]. Previous reviews in Western countries have found strong inverse associations between physical activity and stomach, liver, oesophageal, and breast cancer [2, 3, 31–33]. In addition, several MR studies had validated the causal relationship between physical activity and breast cancer [29]. However, suggestive associations with stomach, liver, oesophageal, and breast cancer were observed in this study as well. This association might be due to the lack of cases and the low intensity of physical activity in this study. MR studies and RCT studies could be conducted in these associations in the future. In addition, previous studies in Western populations have shown a more significant association among non-smokers than smokers [22]. This is consistent with the results in this study. Our study also found that the association was more pronounced among women than men. To date, there is no biological evidence to support a sex difference in the anticancer regimen for physical activity [34, 35].

Previous studies have elucidated several physiological processes through which physical activity influences cancer risk [34–36]. First, higher levels of physical activity are associated with a lower level of insulin-like growth factor (IGF)-1 and fasting glucose and a higher level of insulin-like growth factor-binding protein (IGFBP)-3 [37–39]. This means that physical activity can reduce the likelihood of developing cancer by improving insulin sensitivity and glucose metabolism [40, 41]. Second, previous studies have shown that sex hormones are associated with the development of many types of cancer [42–44]. High levels of physical activity can regulate sex hormone levels by increasing levels of sex hormone-binding globulin (SHBG) [45, 46], ultimately reducing the incidence of cancer. Third, physical activity is associated with lower levels of systemic inflammation by altering inflammatory cytokines or adipokines (e.g., C-reactive protein, adiponectin and interleukin-6) [34, 47, 48], which are associated with a higher risk of cancer [49, 50]. Forth, there are hypotheses that physical activity can reduce the incidence of cancer by affecting oxidative stress [46], DNA methylation and the expression of microRNAs [37]. However, there is a lack of relevant epidemiological evidence for these hypotheses. In addition, pulmonary function is improved by physical activity. Increasing pulmonary ventilation and efficiency shortens lung exposure to carcinogenic substances [51, 52]. Previous studies suggest that higher lung function (measured by Forced Expiratory Volume in One Second (FEV1)) is associated with a lower risk of lung cancer [52]. Meanwhile, physical activity can reduce the risk of colon cancer by increasing vagal tone and decreasing intestinal transit time, thereby reducing the contact time of potential carcinogens such as food residues and bile acids with the colonic mucosa [53].

The present study had some strengths. This was a prospective cohort study for which the association between physical activity and the risk of cancer could be delineated. The conventional potential confounding factors of cancer were adjusted for in the analysis. Data collection and management were performed under rigid quality control. However, the present study had several limitations. First, the assessment of physical activity was self-reported and vulnerable to measurement error. Second, even though separate models were adjusted for multiple established and prospective cancer risk variables, residual confounding by other unmeasured or unknown biological and social factors is still likely. As a result, we are unable to assign causal interpretations to our findings. Third, when malignancies diagnosed within the first two years of follow-up were excluded, most significant relationships were reduced, suggesting that reverse causation bias may have influenced our findings.

## Conclusion

Our findings support that, in total cancer and two of the six cancer sites studied, high levels of physical activity were linked to a decreased chance of developing cancer (lung cancer and colorectal cancer). Additionally, this inverse association between physical activity and total cancer was more pronounced among women and non-smokers. Further studies are required to test this hypothesis.

## Supplementary Information

**Additional file 1:** **Supplementary Table 1.** Coding and cases of cancer outcomes according to the International Classification of Diseases 10th revisions. **Supplementary Table 2.** Associations between daily total physical activity and cancer incidence excluding the first two years of follow-up (excluding 576 participants). **Supplementary Table 3.** Associations between daily total physical activity and cancer incidence excluding those with self-reported poor health (excluding 5,388 participants). **Supplementary Figure 1.** Association between physical activity and cancer type with 95% confidence intervals.

## Data Availability

The datasets used and/or analyzed during the current study are available from the corresponding author on reasonable request.

## Abbreviations

CKB: China Kadoorie Biobank
MET: Metabolic equivalents of tasks
BMI: Body mass index
HR: Hazard ratio
CI: Confidence interval
SD: Standard Deviation
ICD-10: International Statistical Classification of Diseases 10th Revision
