# Supplementary material for: Association between physical activity and cancer risk among Chinese adults: a 10-year prospective study
Source: Int J Behav Nutr Phys Act. 2022 Dec 12;19:150. doi: 10.1186/s12966-022-01390-1 (PMC9743544; doi:10.1186/s12966-022-01390-1)
Supplement: Supplementary file 1 — Additional file 1: Supplementary Table 1. Coding and cases of cancer outcomes according to the International Classification of Diseases 10th revisions. Supplementary Table 2. Associations between daily total physical activity and cancer incidence excluding the first two years of follow-up (excluding 576 participants). Supplementary Table 3. Associations between daily total physical activity and cancer incidence excluding those with self-reported poor health (excluding 5,388 participants). Supplementary Figure 1. Association between physical activity and cancer type with 95% confidence intervals. [file 12966_2022_1390_MOESM1_ESM.docx]

**Supplementary Materials**

**Content**

Supplementary Table 1 Coding and cases of cancer outcomes according to the International Classification of Diseases 10th revisions.

Supplementary Table 2 Associations between daily total physical activity and cancer incidence excluding the first two years of follow-up (excluding 576 participants)

Supplementary Table 3 Associations between daily total physical activity and cancer incidence excluding those with self-reported poor health (excluding 5,388 participants)

Supplementary Figure 1 Association between physical activity and cancer type with 95% confidence intervals

| Supplementary Table 1 Coding and cases of cancer outcomes according to the International Classification of Diseases 10th revisions. | | | |
| --- | --- | --- | --- |
| Cancer site* | ICD-10 codes | Cases |  |
| All cancer | C00-C97 | 3674 |  |
| Oesophagus | C15 | 231 |  |
| Stomach | C16 | 794 |  |
| Colorectal | C18-C20 | 458 |  |
| Liver | C22 | 338 |  |
| Lung | C33-C34 | 722 |  |
| Breast | C50 | 250 |  |
| *: Selecting cancer types with more than 200 cases | | | |

| Supplementary Table 2 Associations between daily total physical activity and cancer incidence excluding the first two years of follow-up (excluding 576 participants) | | | | | | | | | | | | | |
| --- | --- | --- | --- | --- | --- | --- | --- | --- | --- | --- | --- | --- | --- |
| Cause of incidence | | Physical activity | | | | | | | | *P-*trend | | per 1-SD increment | |
|  |  | Q1 | | Q2 | | Q3 | | Q4 | |  |  |  |  |
| Total Cancers | |  | |  | |  | |  | |  | |  | |
| No of events | | 1263 | | 868 | | 694 | | 710 | |  | |  | |
| Model1 | | 1.00 | | 0.96 (0.87, 1.06) | | 0.92 (0.83, 1.03) | | 0.92 (0.82, 1.02) | | 0.084 | | 0.97 (0.93, 1.00) | |
| Model2 | | 1.00 | | 0.96 (0.88, 1.06) | | 0.92 (0.82, 1.02) | | 0.90 (0.81, 1.00) | | 0.033* | | 0.96 (0.92, 1.00) | |
| Colorectal Cancer | |  | |  | |  | |  | |  | |  | |
| No of events | | 175 | | 124 | | 68 | | 75 | |  | |  | |
| Model1 | | 1.00 | | 0.99 (0.77, 1.28) | | 0.67 (0.49, 0.91) | | 0.69 (0.50, 0.95) | | 0.003* | | 0.84 (0.75, 0.94) | |
| Model2 | | 1.00 | | 1.01 (0.78, 1.31) | | 0.68 (0.50, 0.94) | | 0.71 (0.52, 0.98) | | 0.006* | | 0.85 (0.75, 0.95) | |
| Lung Cancer | |  | |  | |  | |  | |  | |  | |
| No of events | | 279 | | 165 | | 121 | | 135 | |  | |  | |
| Model1 | | 1.00 | | 0.90 (0.74, 1.12) | | 0.82 (0.65, 1.04) | | 0.83 (0.65, 1.05) | | 0.086 | | 0.93 (0.85, 1.01) | |
| Model2 | | 1.00 | | 0.89 (0.72, 1.10) | | 0.79 (0.62, 1.00) | | 0.78 (0.61, 0.99) | | 0.028* | | 0.91 (0.83, 0.99) | |
| Multivariate models were stratified by age (5-year intervals) and adjusted for: Model 1: sex; Model 2: additionally adjusted for education, marital status, alcohol intake, smoking status, fresh fruit consumption, red meat consumption, family histories of cancer and BMI.  *: *P*< 0.05 | | | | | | | | | | | | | |

| Supplementary Table 3 Associations between daily total physical activity and cancer incidence excluding those with self-reported poor health (excluding 5,388 participants) | | | | | | | | | | | | | |
| --- | --- | --- | --- | --- | --- | --- | --- | --- | --- | --- | --- | --- | --- |
| Cause of incidence | | Physical activity | | | | | | | | *P-*trend | | per 1-SD increment | |
|  |  | Q1 | | Q2 | | Q3 | | Q4 | |  |  |  |  |
| Total Cancers | |  | |  | |  | |  | |  | |  | |
| No of events | | 1052 | | 761 | | 637 | | 651 | |  | |  | |
| Model1 | | 1.00 | | 0.97 (0.88, 1.07) | | 0.92 (0.83, 1.03) | | 0.94 (0.85, 1.05) | | 0.172 | | 0.97 (0.94, 1.01) | |
| Model2 | | 1.00 | | 0.97 (0.88, 1.07) | | 0.91 (0.82, 1.01) | | 0.92 (0.82, 1.02) | | 0.058 | | 0.96 (0.93, 1.00) | |
| Colorectal Cancer | |  | |  | |  | |  | |  | |  | |
| No of events | | 151 | | 113 | | 64 | | 70 | |  | |  | |
| Model1 | | 1.00 | | 1.01 (0.79, 1.30) | | 0.66 (0.48, 0.90) | | 0.72 (0.53, 0.97) | | 0.002* | | 0.84 (0.75, 0.94) | |
| Model2 | | 1.00 | | 1.02 (0.79, 1.31) | | 0.67 (0.49, 0.91) | | 0.72 (0.53, 0.99) | | 0.003* | | 0.84 (0.75, 0.94) | |
| Lung Cancer | |  | |  | |  | |  | |  | |  | |
| No of events | | 235 | | 148 | | 109 | | 122 | |  | |  | |
| Model1 | | 1.00 | | 0.91 (0.74, 1.13) | | 0.76 (0.60, 0.97) | | 0.82 (0.64, 1.03) | | 0.038* | | 0.91 (0.84, 0.99) | |
| Model2 | | 1.00 | | 0.89 (0.72, 1.10) | | 0.73 (0.57, 0.93) | | 0.76 (0.60, 0.97) | | 0.010* | | 0.89 (0.82, 0.97) | |
| Multivariate models were stratified by age (5-year intervals) and adjusted for: Model 1: sex; Model 2: additionally adjusted for education, marital status, alcohol intake, smoking status, fresh fruit consumption, red meat consumption, family histories of cancer and BMI.  *: *P*< 0.05 | | | | | | | | | | | | | |


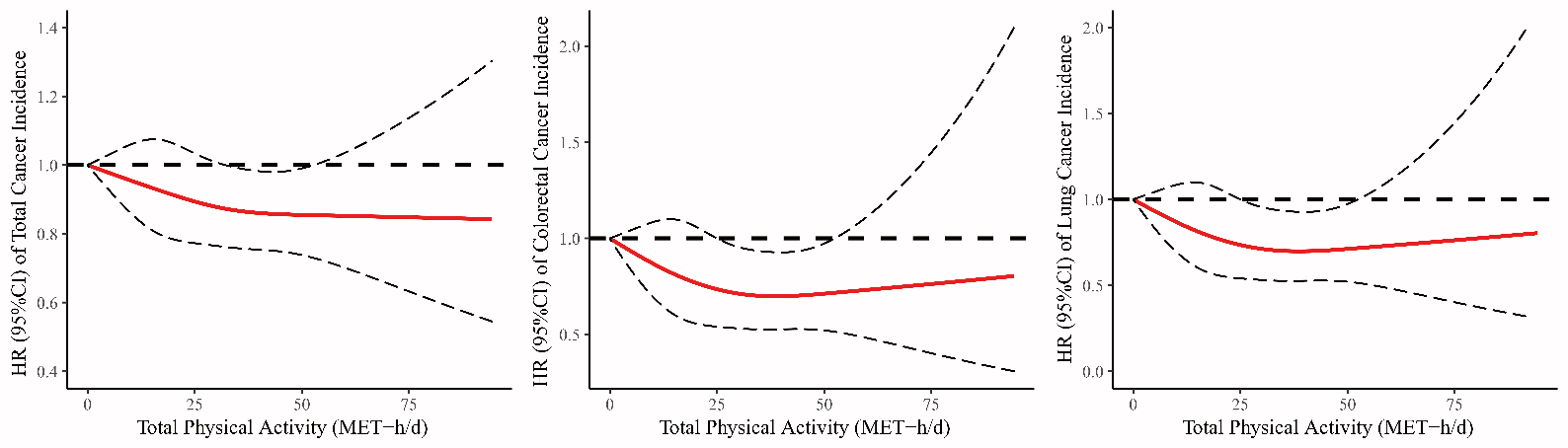
Supplementary Figure 1 Association between physical activity and cancer type with 95% confidence intervals
